# Rethinking Mitral Annular Calcification and Its Clinical Significance: From Passive Process to Active Pathology

**DOI:** 10.3390/jpm14090900

**Published:** 2024-08-25

**Authors:** Paula Cristina Morariu, Alexandru Florinel Oancea, Evelina Maria Gosav, Oana Nicoleta Buliga-Finis, Magdalena Cuciureanu, Dragos-Viorel Scripcariu, Oana Sirbu, Maria Mihaela Godun, Diana-Elena Floria, Petronela Cristina Chiriac, Livia Genoveva Baroi, Anca Ouatu, Daniela Maria Tanase, Ciprian Rezus, Mariana Floria

**Affiliations:** 1 Department of Internal Medicine, “Grigore T. Popa” University of Medicine and Pharmacy, 700115 Iasi, Romania; morariu.paula-cristina@email.umfiasi.ro (P.C.M.); evelina.maria.gosav@umfiasi.ro (E.M.G.); oana-nicoleta.buliga-finis@umfiasi.ro (O.N.B.-F.); oana.sirbu@umfiasi.ro (O.S.); godun.maria-mihaela@email.umfiasi.ro (M.M.G.); diana-elena.iov@d.umfiasi.ro (D.-E.F.); anca.ouatu@umfiasi.ro (A.O.); daniela.tanase@umfiasi.ro (D.M.T.); ciprian.rezus@umfiasi.ro (C.R.); floria.mariana@umfiasi.ro (M.F.); 2 Internal Medicine Clinic, “St. Spiridon” County Clinical Emergency Hospital Iasi, 700111 Iasi, Romania; chiriac.cristina@spitalspiridon.ro; 3 Cardiology Clinic, “Sf. Spiridon” Emergency Hospital Iasi, 700111 Iasi, Romania; 4 Department of Pharmacology, “Grigore T. Popa” University of Medicine and Pharmacy, 700115 Iasi, Romania; mag.cuciureanu@umfiasi.ro; 5 Regional Institute of Oncology, 700483 Iasi, Romania; dragos-viorel.scripcariu@umfiasi.ro; 6 Institute of Gastroenterology and Hepatology, “St. Spiridon” County Clinical Emergency Hospital, 700111 Iasi, Romania; 7 Department of General Surgery, Faculty of Medicine, “Grigore T. Popa” University of Medicine and Pharmacy, 700115 Iasi, Romania

**Keywords:** mitral annular calcification, atherosclerosis, valve disease, aging, inflammation, metabolic syndrome, stroke

## Abstract

Background: Mitral annulus calcification is a chronic degenerative condition affecting the fibrous base of the mitral valve. Historically viewed as an age-related phenomenon, recent studies suggest it is driven by active mechanisms involving systemic inflammation, hemodynamic stress, abnormal calcium-phosphorus metabolism, and lipid accumulation. Despite often being asymptomatic and incidentally detected, its clinical relevance stems from its strong association with increased cardiovascular disease risk, higher cardiovascular mortality, and elevated overall mortality. Methods: This article investigates the complexities and controversies surrounding mitral annular calcification as a potential embolic source, focusing on its diagnosis, its relationship with systemic inflammation, and its links to metabolic and chronic disorders. Results: The findings highlight that mitral annular calcification is not merely a passive marker of aging but an active indicator of atherosclerotic burden with significant implications for cardiovascular health. Conclusion: Mitral annulus calcification should be recognized as an important factor in cardiovascular risk assessment, offering insight into systemic inflammatory processes and metabolic dysregulation.

## 1. Introduction

Mitral annular calcification (MAC) results from the progressive calcification of the fibrous mitral annulus, primarily affecting the posterior part of the annulus. Originally considered a process linked primarily to aging, recent research reveals that MAC involves active mechanisms such as inflammation, hemodynamic stress, lipid deposition, and new bone formation [1]. MAC is commonly observed, with its prevalence increasing with age, reaching up to 10% in individuals over 60 years old. The presence of MAC varies by sex, and the few studies on these differences that are available reveal that women had more extensive MAC [1,2]. MAC occurs frequently across all major racial and ethnic groups and does not show a significant association with ethnic ancestry [1,3].

Epidemiologic studies have shown a strong association between MAC and cardiovascular risk factors, leading to the hypothesis that MAC may serve as a marker of atherosclerotic burden. It is often found in conjunction with other atherosclerotic risk factors, including tobacco use, hypertension (HTN), chronic kidney disease (CKD), obesity, dyslipidemia, and Type 2 diabetes (T2D) [4,5]. Consequently, MAC has been consistently linked to increased cardiovascular mortality and morbidity, including myocardial infarction and stroke [6].

This article delves into the multifaceted and contentious issues surrounding MAC as a potential source of emboli. It provides an in-depth examination of the various complexities involved in diagnosing MAC and its intricate relationship with systemic inflammation. Additionally, the article investigates the associations between MAC and various metabolic disorders, highlighting the nuanced ways in which these factors interrelate. By addressing these aspects, the article aims to shed light on the broader implications of MAC in clinical practice and its impact on patient health.

## 2. Mitral Annular Calcification: From Definition to Diagnosis and Assessment

The mitral annulus serves as the boundary between the left atrium (LA) and the left ventricle (LV). It is typically divided into anterior and posterior sections. The anterior annulus stretches between the left and right fibrous trigones and is anatomically connected to the aortic annulus, forming what is known as the aortomitral curtain. The posterior annulus covers the remaining annular perimeter and consists of a discontinuous rim of fibrous tissue interspersed with fat. This composition is believed to make the posterior annulus particularly prone to remodeling and enlargement. Externally, the posterior annulus is related to the musculature of the LV inflow region, and internally, it is associated with the LA, merging with the hinge of the posterior mitral leaflet [7]. The mitral annulus is crucial for leaflet coaptation, mitigating mitral valve-closing forces, and facilitating the filling and emptying of the LA and LV. Disruptions in the geometry and mechanics of the annulus are key factors in several conditions, including mitral valve prolapse, MAC, mitral regurgitation (MR), atrial fibrillation (AF), or annular sub-mitral aneurysm [7].

MAC is described by calcium formation along the annulus, primarily affecting the posterior part of the annulus, and it may progress to the posterior leaflet or involve the anterior part as the severity increases. First considered as a consequence of chronic degeneration associated with aging, MAC is now largely understood as an actively controlled molecular process involving injuries, lipid deposits, inflammation, and bone formation. MAC is frequently encountered in cardiovascular imaging studies, surgical procedures, and post-mortem examinations [8].

Using multimodal imaging techniques such as 2D and 3D echocardiography, Doppler echocardiography, and cardiac computed tomography (CT) can precisely determine the extent and location of MAC, aiding in the development of effective treatment strategies [9]. Transthoracic echocardiography (TTE) is the main imaging technique used to examine the mitral valve’s structure and function, as well as to diagnose MAC. Recently, CT has also become crucial for assessing the mitral valve, particularly for detecting MAC [10].

**Figure 1:**
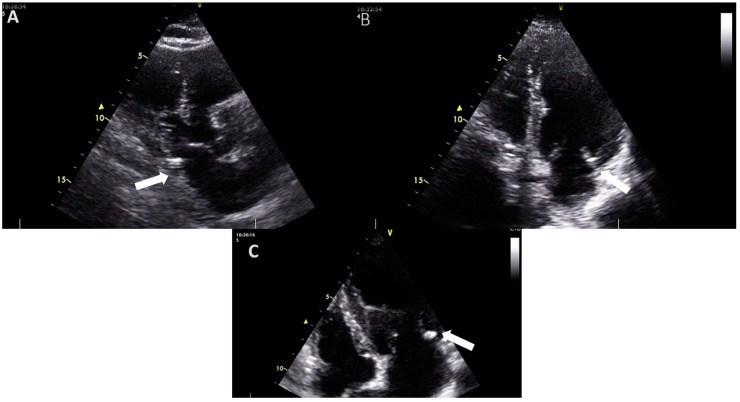
The appearance of mitral annular calcification on transthoracic echocardiography ((**A**), transthoracic parasternal long axis view (**B**), and (**C**) transthoracic apical 4-chamber view). It appears as an echogenic structure at the base of the posterior leaflet (white arrow). Images are from the personal collection of Professor Floria Mariana.

An echocardiographic examination of the mitral valve involves assessing the mitral annulus, leaflets, and sub-valvular apparatus to detect and evaluate the presence, location, and extent of calcifications [10]. On echocardiography, MAC appears as a dense, shell-like structure, usually located under the posterior leaflet and parallel to the free wall of the left ventricle (Figure 1). This is typically seen with associated acoustic shadowing in the parasternal long axis, parasternal short axis, and apical views [5,8,10]. MAC can also be present in the medial segment of the anterior leaflet [5].

**Figure 2:**
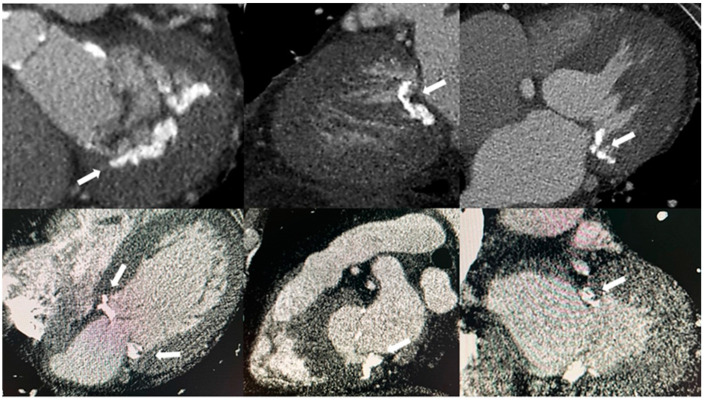
The appearance of mitral annular calcification on computed tomography (white arrows).

CT scans can visualize MAC without using additional contrast agents. An ECG-gated cardiac CT scan can further help determine the precise location and extent of MAC when cardiac catheterization and TTE do not provide a clear enough description of it [5,8,11]. This examination pinpoints the most heavily calcified areas, and their extent assesses the feasibility of suturing during surgery and provides crucial information about the risk of complications during the procedure [5]. To evaluate MAC effectively, CT scans should ideally provide high-resolution images of the junction between the LA and LV. This allows for a clear assessment of the disease’s full extent, which can sometimes spread into the LV, interventricular septum, and right ventricle [10]. Additionally, a CT scan (Figure 2) most accurately identifies the location of the calcification in the mitral valve apparatus and distinguishes it from calcium deposits in the aortic valve or coronary arteries [8].

Furthermore, based on CT evaluations, Guerrero et al. proposed a scoring system to assess the severity of MAC. This system considers factors such as calcium thickness, calcium distribution around the annular circumference, calcification of the trigones, and calcification of the anterior mitral leaflet. The severity of MAC is then determined by the total points accumulated from these characteristics [12,13].

Chest radiography has been utilized for approximately a century to evaluate MAC. Calcification in the mitral annulus appears as a C, J, or U shape, with the open part of this incomplete circle located at the aortic outflow tract, positioned above and in front of the calcification [5,14].

Evaluating calcification like MAC is difficult with cardiac magnetic resonance (CMR) compared to other methods, limiting its role. However, CMR can assess caseous calcifications, showing a hypointense rim and hyperintense center on T1-weighted images, and the opposite on T2-weighted images. CMR is useful for accurately measuring chamber size, function, flow, and regurgitation, aiding in quantifying MR associated with MAC [10].

Nuclear imaging can help assess calcification and inflammation in MAC. One study conducted by Massera et al. used 18F-sodium fluoride and 18F-fluorodeoxyglucose PET to detect these activities [4]. Reducing inflammation might slow calcification, offering potential treatment options for MAC. More research on the use of nuclear imaging for MAC is needed. However, access and radiation exposure are limitations of this method [4,10].

Currently, there is no universally accepted classification system for the severity of MAC. However, in some studies, MAC has been characterized as mild (characterized by focal, soft echogenicity of the mitral annulus, covering less than 180° of the total annular circumference), moderate (displaying more noticeable echogenicity of the annulus, covering less than 270° of the total annular circumference), or severe (exhibiting marked echo-density encompassing more than 270° of the mitral annulus circumference or extending into the left ventricular inflow tract, with a thickness exceeding 4 mm) [5,15].

CT-based techniques show promise due to their superior spatial resolution for calcification. Like the grading system for TTE, a CT scan can qualitatively assess MAC. The Agatston method, which quantifies calcification by a weighted sum of Hounsfield units from non-contrast axial images, can be adapted to MAC. A study of 559 patients with severe aortic stenosis found a high inter-reader agreement for no MAC and severe MAC (k = 0.88 and k = 0.75, respectively) using a semi-quantitative method, but only moderate agreement for mild and moderate MAC (k = 0.45 and k = 0.59, respectively). Intra-reader agreement was also moderate (k = 0.69 and 0.62). In contrast, the Agatston method provided reproducible results with high inter- and intra-reader agreement (intra-class correlation coefficients of 0.998 and 0.999, respectively) [16].

Caseous calcification of the mitral annulus (CCMA) is a relatively rare development stemming from MAC, with prevalence estimated to be approximately 0.6% among patients diagnosed with MAC [5,17,18,19]. The exact mechanism is unclear, and most patients are asymptomatic, though CCMA can occasionally cause significant issues. On TTE, CCMA appears as a circular, echo-dense mass in the periannular region, typically near the posterior leaflet [18,19]. It lacks acoustic shadowing artifacts and contains central areas with echo-lucencies resembling liquefaction. It can be mistaken for cardiac tumors or abscesses and can coexist with endocarditis [19,20,21]. Diagnosing CCMA may be challenging with TTE alone, but cardiac CT can help, showing it as an oval or crescent-shaped hyperdense mass with a calcified rim [5,17,18,19].

## 3. Mitral Annular Calcification and Cardiovascular Diseases

MAC is a chronic degenerative condition that worsens with age and can lead to mitral stenosis (MS), MR, heart failure, infective endocarditis, atrial arrhythmias, and heart blocks. Additionally, higher rates of MAC are observed in patients with systemic HTN, increased stress on the mitral valve, mitral valve prolapse, elevated LV systolic pressure, aortic valve stenosis, and AF. It is also linked to a higher prevalence of risk factors for coronary atherosclerosis [1,15,22].

Multiple studies have demonstrated that MAC is an independent predictor of both all-cause and cardiovascular mortality [8,22]. For instance, the Framingham Heart Study found that individuals with MAC had a 60% higher risk of cardiovascular death and a 30% higher risk of all-cause mortality after adjustments [23]. Similarly, the Cardiovascular Health Study (CHS) in an older population reported an 80% increase in cardiovascular death risk and a 30% increase in all-cause death risk associated with MAC [24]. The ARIC study also highlighted that MAC was linked to a 132% higher risk of coronary heart disease events, even after accounting for other risk factors [25].

### 3.1. Mitral Valve Disfunction

MAC is a degenerative condition where calcium deposits form around the mitral valve’s annulus, leading to various forms of mitral valve dysfunction (MVD) like MR, MS, or mixed valve disease [26].

Frequently, MR coexists with MAC, with an association rate ranging from 15% to 55% [5]. MR associated with MAC occurs due to the impaired sphincter-like function of the mitral annulus during systole or from the disruption of leaflet coaptation caused by MAC-induced deformation [8,27]. Additionally, cases of chordal rupture due to the mechanical stress exerted by MAC have also been reported [7]. MS can also develop as a result of severe MAC [28]. This typically occurs when MAC leads to a significant reduction in the effective orifice area and restricts the opening of the posterior leaflet [29]. The likely underlying mechanism involves microinjury and endothelial dysfunction, exacerbated by increased stress on the valve’s fibrous skeleton. Due to these factors, it is plausible to consider MS more as a result of ongoing damage than as an initial cause of the condition [5].

The progression of MAC and how it leads to MVD is not well understood [6]. One recent study aimed to track how MAC progresses from mild to moderate to severe and to observe how this progression leads to MVD [6]. Out of 11,605 patients, 78% had mild MAC, 17% moderate, and 5% severe MAC. Follow-up TTE showed that 33% of patients with mild or moderate MAC developed severe MAC within 10 years. Women had a higher progression rate to severe MAC than men (41% vs. 24%; *p* < 0.001), and patients with moderate MAC progressed faster than those with mild MAC (71% vs. 22%; *p* < 0.001). After 10 years, 10% of patients had MVD, with prevalence increasing from 4% in mild MAC to 60% in severe MAC. Female sex was a strong predictor of MVD (15% vs. 5%; *p* < 0.0001) [6]. Another study also found that the progression of MAC is common and linked to structural changes and increased hemodynamic stress, leading to mechanical stress [30]. Furthermore, patients with advancing MAC tend to have worse outcomes [30].

The relationship between MAC and MVD is critically important because MAC significantly influences the approach to treating MVD. Performing mitral valve surgery in patients with MAC carries a high risk due to factors like advanced age, the frequent presence of multiple comorbid conditions, and the technical difficulties posed by calcification, which may require extensive debridement or reconstruction of the mitral annulus [26,31,32,33]. As a result, surgical intervention for MVD in the context of MAC is often postponed until symptoms become severe, or a more conservative treatment strategy is chosen instead [31,34,35]. Effectively managing MAC with accompanying MVD necessitates a multidisciplinary approach. This strategy carefully weighs the risks and benefits of surgical versus transcatheter interventions, considering the patient’s overall health and the degree of calcification [36].

### 3.2. Coronary Artery Disease

Experts classify MAC as a type of atherosclerosis, recognizing its correlations with other atherosclerotic conditions such as aortic atheroma, carotid artery disease, peripheral vascular disease, and coronary artery disease (CAD) [37]. These associations were initially identified around 30 years ago using less precise imaging techniques for investigation. However, recent studies are now challenging these earlier findings [37]. For example, a cross-sectional study published in 2024 aimed to explore the clinical and prognostic significance of MAC in relation to CAD using coronary CT angiography. The study included 205 participants, with 85 having MAC and 120 without. CAD severity was assessed using the CAD-RADS system, classifying participants into no or non-significant CAD (CAD-RADS 0–2) and significant CAD (CAD-RADS 3–5). Patients with MAC had higher coronary artery calcification scores (*p* = 0.05). While MAC initially showed a significant association with severe CAD (OR 1.96, 95% CI 1.04–3.71, *p* = 0.04), this association lost significance after adjusting for confounders (OR 1.60, 95% CI 0.78–3.28, *p* = 0.2). Overall, the study found that MAC does not provide independent prognostic value for coronary atherosclerosis when evaluated using coronary CT angiography [37].

However, recent studies often contradict Morandi et al.’s findings, suggesting a significant link between MAC and CAD, likely due to their shared underlying causes. For instance, another recent study included patients who were undergoing coronary angiography [38]. Their findings included: 66% of MAC patients had single-vessel disease (SVD), compared to 33% without MAC; 68% of MAC patients had double-vessel disease (DVD), versus 32% without MAC; 83% of MAC patients had triple-vessel disease (TVD), compared to 17% without MAC, and all patients with MAC had left main coronary artery disease, whereas 88% of no-MAC patients had no significant disease. More than this, multivariate analysis identified MAC (*p* = 0.007) as an independent predictor of CAD even for patients under 60 years old [38]. Another research was performed using the KOrea Initiatives on Coronary Artery (KOICA) registry [39]. They analyzed 722 asymptomatic individuals who had serial cardiac CT scans and measured MAC and calcification of the coronary arteries on CT and quantified their severity using Agatston units (AU). Greater coronary artery calcification severity was linked to a higher likelihood of having MAC (OR per 100 AU increase: 1.06, 95% CI 1.05–1.07, *p* < 0.001), even after adjusting for baseline characteristics [39]. Overall, while MAC and CAD are related through common risk factors and possible shared pathophysiological mechanisms, the precise nature of their relationship requires more investigation to be fully understood. Due to the ongoing debate and mixed findings, further research is needed to better understand the relationship between MAC and CAD, particularly to establish if MAC can reliably predict CAD or if it merely reflects shared risk factors.

## 4. Mitral Annular Calcification and Its Role in Stroke Risk

The first documented case of a stroke in a patient with MAC was reported by Rytand et al. in 1946. Since then, numerous case reports and clinical studies have explored the potential link between stroke and MAC [40,41,42,43,44,45,46].

A recent study followed patients with MAC for 15 years, using cardiac CT to assess the extent of calcification. After adjusting for various factors including age, sex, race, smoking, blood pressure, diabetes, fibrinogen, high-sensitivity C-reactive protein (hs-CRP), IL-6, and coronary artery calcium score, MAC was linked to a higher risk of all types of strokes. Furthermore, MAC continued to be a strong predictor of stroke even when AF or atrial flutter (AFL) and left atrial (LA) dimensions were present. The study concluded that MAC is an independent long-term predictor of stroke risk, regardless of common cardiovascular risk factors and the presence of AF [47]. Significant studies, including the LIFE study, the MESA study, the Strong Heart Study, and the Framingham Heart Study, have all reported similar findings, highlighting the increased risk of stroke in individuals with MAC [48,49,50,51].

Several mechanisms have been tried to explain the involvement of MAC in stroke risk. MAC could be a marker of subclinical LA fibrosis or dysfunction, potentially increasing LA thrombus formation risk. It remains unclear if anticoagulation or antiplatelets reduce stroke risk in MAC patients. The Framingham study found MAC doubled the stroke risk, even without AF. Preventing MAC formation and progression might also reduce stroke risk [49,51].

MAC might not solely indicate an increased risk of stroke but could also actively contribute to thromboembolism [50]. Several reports support this idea by describing instances of mobile calcific or thrombotic debris, often in cases where there is clear ulceration of annular calcium, in patients with cerebral embolism. Some reports have even documented calcific emboli in the artery related to the infarct during autopsy [50]. Furthermore, MAC may heighten the likelihood of developing infective endocarditis by providing a site for infective vegetation to form and then increasing the risk of embolization of vegetative material. Although the exact mechanisms are unclear, several potential explanations exist. Histopathologic examination has shown endocardial inflammation and ulceration associated with annular calcium, leading to platelet aggregation and thrombus formation, which are essential for vegetation formation. Additionally, large calcific deposits can distort the annulus and alter flow patterns, promoting bacterial deposition in the annular region [46,51,52,53,54].

Even if, to date, no definitive link between ischemic stroke and MAC has been established, the European Association of Cardiovascular Imaging (EACVI) included MAC as a potential source of embolism along with other conditions like calcified aortic stenosis, patent foramen ovale, Giant Lambl’s excrescences, and mitral valve prolapse [9,55]. The likelihood of an embolic stroke is greater when massive MAC is present [55]. The Framingham Heart Study found that for every millimeter increase in MAC thickness measured by M-mode echocardiography, the relative risk for stroke increased by 1.24 [49,56]. However, more recent studies have found that it is not MAC thickness that is associated with stroke, but rather other complex features of MAC, like mobility, suggestive aspect CCMA, or functional MS [56]. Functional MS can raise LA pressure, which in turn increases the likelihood of thrombus formation, explaining its role in the occurrence of stroke [56]. While some case reports suggest a potential link between CCMA and stroke, the exact mechanisms—such as small calcific emboli, thrombus formation from surface ulceration, or direct fistulization—are not well understood. More research, including multimodal imaging studies, is needed to clarify the clinical significance of CCMA [56].

The thromboembolic (TE) risk can be evaluated, especially in patients with AF, using the CHA_2_DS_2_-VASc score and its earlier version, CHADS_2_. However, the connection between MAC and these scoring systems has not been firmly established yet. Several studies have examined TE risk scores in MAC patients (with or without AF) and have found higher scores in these patients compared to those without MAC. However, additional large-scale studies are required to determine the precise predictive value of the CHA_2_DS_2_-VASc and CHADS_2_ scores in patients with MAC [57,58,59].

## 5. Mitral Annular Calcification: A Marker of Systemic Inflammation

MAC has been proposed as an indicator of atherosclerosis. This is not unexpected, since MAC and atherosclerosis have common clinical risk factors for cardiovascular diseases (CVD), such as age, obesity, hyperlipidemia, diabetes mellitus, and arterial HTN [60,61]. Numerous studies have demonstrated that MAC is linked to atherosclerotic risk factors and various types of atherosclerotic CVD, including carotid artery stenosis, CAD, and aortic atherosclerosis [61,62]. MAC is often considered a form of atherosclerotic heart disease due to shared risk factors and underlying atherosclerosis. Risk factors for MAC, such as age, diabetes, HTN, and obesity, may lead to inflammation [60].

Previous research indicates that MAC could be considered a type of atherosclerosis. Due to the strong link between systemic inflammation and atherosclerosis, inflammation might play a role in signaling the development of MAC. Nonetheless, there is limited data on the connection between MAC and systemic inflammation [60,61].

It has been established that the early stages of atherosclerosis involve the attachment of leukocytes to the endothelial cells lining the arteries, facilitated by adhesion molecules like intracellular adhesion molecule-1 (ICAM-1) and vascular cell adhesion molecule-1 (VCAM-1). This leukocyte recruitment is an early marker of inflammation triggered by hyperlipidemia. As atherosclerosis develops, it involves an ongoing inflammatory response, with various immune cells like macrophages and T cells playing key roles [63,64]. Once the inflammatory cascade is activated, molecules such as IL-1β and IL-6 are produced, and C-reactive protein (CRP) production is stimulated [63,64]. Furthermore, proinflammatory cytokines from activated macrophages in atherosclerotic plaques cause vascular smooth muscle cell apoptosis, releasing calcium- and phosphate-rich vesicles that initiate calcium deposition [64].

The preponderance of evidence strongly indicates a significant link between the calcification of the fibrous base of the heart, which includes the mitral and aortic annuli, and changes related to vascular atherosclerosis. Experimental induction of systemic arterial atherosclerosis leads to the development of fatty plaques on both the aortic surface of the aortic valve cusps and the ventricular surface of the posterior mitral leaflet. As these fatty plaques enlarge, they experience nutrient deficiency and subsequently undergo transformation into calcific deposits [65]. This conclusion is supported by pathological studies that reveal the presence of foam cell accumulations on the endothelium of the epicardial coronary arteries, the ventricular surface of the posterior mitral leaflet, and the aortic aspects of each aortic valve cusp, detectable as early as adolescence and persisting into the second and third decades of life. These findings suggest that the presence of MAC likely indicates a systemic atherosclerotic process involving not only the aortic and mitral valves but also the coronary arteries and potentially other arterial systems [65].

### 5.1. C-Reactive Protein and High-Sensitivity C-Reactive Protein

Several studies have documented an association between atherosclerosis and inflammation. Elevated levels of various inflammatory markers, such as CRP, have been linked to a higher risk of developing atherosclerotic disease [61].

In a study of 100 patients diagnosed with MAC, Kurtoglu et al. noted elevated levels of hs-CRP compared to a control group. These elevated hs-CRP levels persisted among MAC patients, regardless of whether they had HTN or CAD [65]. However, researchers note that their study does not sufficiently explain a cause-and-effect relationship, and it remains unclear whether MAC leads to increased hs-CRP levels, if elevated CRP levels precede the development of MAC, or if other factors influence these conditions [65]. These results are similar to those of another study, the Cardiovascular Health Study, where participants with MAC exhibited higher serum levels of CRP compared to those without MAC [24], reinforcing the idea that inflammation is present in patients with MAC.

However, findings regarding CRP levels and their association with MAC vary. In the MESA study, CRP levels did not show a significant increase in patients with MAC after adjusting for all cardiovascular risk factors [61]. This can be attributed to the fact this can be attributed to the fact that the development of MAC, in addition to potentially being linked to an underlying atherosclerotic process, also involves a chronic degenerative, non-inflammatory process characterized by calcification of the fibrous support around the mitral valve [61].

### 5.2. Neutrophil–Lymphocyte Ratio

White blood cell (WBC) count independently predicts CVD and all-cause mortality, and it may identify high-risk individuals who are not detected by traditional CVD risk factors [66]. In the GRACE registry, leukocyte count on admission in 8269 patients was related to in-hospital death (adjusted odds ratio [OR] 2.8, 95% CI 2.1–3.6) and heart failure (OR 2.7, 95% CI 2.2–3.4) in people presenting with acute coronary syndrome. Leukocytes and, more specifically, neutrophils have a key role in both atherogenesis [66,67].

The neutrophil–lymphocyte ratio (NLR) is easy to calculate, accessible, available in any laboratory, inexpensive, and non-invasive. This marker has been proven useful for assessing systemic inflammation [60,66].

The relationship between CVD and NLR is not yet fully understood. However, factors such as inflammation, endothelial dysfunction, and oxidative stress appear to play a role. Neutrophils can damage the endothelium through their interactions with this tissue. They promote the secretion of inflammatory mediators and the release of proteolytic enzymes and superoxide radicals, all of which are crucial in the atherosclerosis process [60,68].

The NLR correlates with the severity of CAD and is also linked to the angiographic progression of coronary atherosclerosis [60,67]. It serves as an independent predictor of unfavorable outcomes in patients with ST-segment elevation myocardial infarction who underwent percutaneous coronary intervention [60]. Studies have consistently demonstrated that a greater NLR is independently linked to arterial stiffness and the coronary calcium score. Moreover, NLR has been found to be correlated to coronary and thoracic peri-aortic calcification in patients with end-stage renal disease [60].

NLR has been identified as a predictor of atrial fibrillation, and a high NLR is linked to a greater risk of recurrence of the condition. The NLR value has also been linked to heart failure, which is associated with higher levels of inflammation. An elevated NLR is connected to a greater risk of long-term mortality in these patients [69]. In valvular heart disease, patients with severe mitral stenosis had higher NLR levels compared to those with moderate or mild mitral stenosis. Similarly, patients with severe aortic stenosis and preserved ejection fraction also showed significantly higher NLR levels than those with mild or moderate aortic stenosis [69]. Additionally, NLR has been linked to HTN, metabolic syndrome (MS), and diabetes, which are risk factors for both atherosclerosis and MAC [70].

The relationship between NLR and MAC is still being studied, with only a few research findings showing the connection between them. Varol et al. included 117 patients diagnosed with MAC in their study. They found that neutrophil counts were significantly higher in these patients compared to the control group (5.59 ± 2.13 vs. 3.59 ± 1.02 × 10^3^/mL; *p* < 0.001). In contrast, lymphocyte counts were lower in the MAC group (1.88 ± 0.68 vs. 2.20 ± 0.49 × 10^3^/mL; *p* = 0.01) [60]. As a result, NLR was significantly higher in MAC patients (3.3 ± 1.8 vs. 1.6 ± 0.4; *p* < 0.001), with a positive correlation to MAC (*p* < 0.001, r = 0.58) [60]. Additionally, WBC counts were higher in MAC patients, and this difference was statistically significant (8.45 ± 2.52 vs. 6.48 ± 1.43 × 10^3^ mg/mL; *p* < 0.001) [60]. They also observed significant increases in red cell distribution width (RDW) (16.2 ± 3.3 vs. 13.4 ± 0.9%; *p* < 0.001) and eosinophil counts (0.20 ± 0.19 vs. 0.10 ± 0.07 × 10^3^/mL; *p* = 0.001) [60].

Another hematologic marker linked to inflammation and atherosclerosis is the platelet-to-lymphocyte ratio (PLR). In a study involving 1060 patients, researchers examined the relationship between PLR and MAC. They found that PLR was significantly higher in the MAC patients compared to the control group (129.1 ± 32.2 vs. 103.5 ± 23.8; *p* < 0.001), and an elevated PLR was independently associated with the presence of MAC. The study also evaluated NLR in these patients and found significantly higher levels in the study group, which were also independently linked to MAC (OR: 1.328, 95% CI: 1.038–1.699; *p* = 0.024) [62].

### 5.3. Fetuin-A

Fetuin-A, also known as α2-Heremans-Schmid-glycoprotein, is a glycoprotein that is primarily produced in the liver, adipose tissue, tongue, and placenta [71]. It is a member of the cystatin superfamily of cysteine protease inhibitors and plays various roles in the body [72]. Fetuin-A has been found to play a role in numerous biological processes, sparking a significant increase in interest recently. These processes include:

1. Mineralization inhibition: Fetuin-A is a potent inhibitor of pathological calcification. It binds to calcium and phosphate, preventing the formation of insoluble calcium phosphate crystals that can lead to vascular calcification and other mineralization disorders. Conversely, low levels of Fetuin-A were linked to an increase in systemic vascular calcifications and cardiovascular comorbidities [72,73,74,75,76].
2. Atherogenic effects: Fetuin-A levels are associated with carotid arterial stiffness, and lower levels of this protein are linked to increased formation of atherosclerotic plaques. This relationship contributes to the progression of CVD [77,78,79].
3. Inflammatory and anti-inflammatory effects: Fetuin-A can have either anti-inflammatory or pro-inflammatory effects, depending on how it is activated in different pathological conditions. It is an acute-phase negative protein, inversely related to CRP levels, as it reduces its hepatic production in response to circulating inflammatory cytokines. Fetuin-A displays anti-inflammatory properties by regulating the immune response and inhibiting tumor necrosis factor-α (TNF-α) and transforming growth factor-β (TGF-β), playing a beneficial role in various clinical conditions, including sepsis, Crohn’s disease, ulcerative colitis, chronic obstructive pulmonary disease (COPD), rheumatoid arthritis, and brain disorders [76,80,81,82,83,84,85].
4. Metabolic regulation: Fetuin-A has been implicated in metabolic processes, including insulin resistance and the development of T2D. Higher levels of fetuin-A are associated with obesity, MS, and fatty liver disease [78,80,86,87].

Small studies have revealed a connection between MAC and fetuin-A [88]. In a study involving 970 patients with CAD, Ix et al. found that fetuin-A levels were inversely related to MAC in patients without chronic kidney disease (CKD). Additionally, they observed that fetuin-A levels were also low in patients with aortic stenosis who did not have T2D [89]. Another study aimed to evaluate the predictive value of serum fetuin-A and MAC in patients with suspected or diagnosed CAD. It involved 54 patients without renal impairment or rheumatic valvular disease and concluded that low fetuin-A levels were associated with MAC. Additionally, fetuin-A levels were negatively correlated with hs-CRP and LDL cholesterol (LDL-c) [90].

In the Cardiovascular Health Study, the researchers analyzed data from 3585 participants focusing on biomarkers associated with lipid metabolism (such as lipoprotein (Lp)(a) and LpPLA2), inflammation (including IL-6 and soluble CD14), and mineral metabolism (like fetuin-A and fibroblast growth factor (FGF-23). Fetuin-A was negatively associated with MAC, highlighting once again the role of fetuin-A in valve calcification [88]. However, more extensive studies are needed to confirm the relationship between these two factors.

## 6. Mitral Annular Calcification across Metabolic and Chronic Diseases

**Figure 3:**
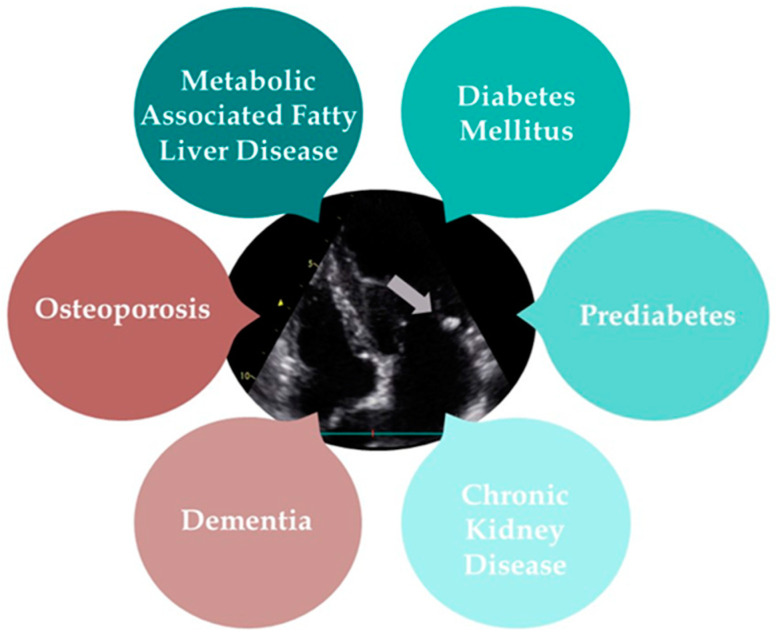
The multisystem impact of mitral annular calcification (grey arrow).

MAC is a type of heart valve calcification that has been increasingly linked to a range of systemic diseases. Metabolic-associated fatty liver disease (MAFLD), T2D, prediabetes, CKD, osteoporosis, and dementia all demonstrate significant associations with MAC, highlighting its role as a marker for broader health issues. The following text will provide a detailed overview of the diseases commonly associated with MAC (Figure 3).

### 6.1. Metabolic-Associated Fatty Liver Disease

Nonalcoholic fatty liver disease (NAFLD) is a multisystem condition with various extrahepatic manifestations, including obesity, T2D, MS, CVD, CKD, and malignancies [91]. Previously known as NAFLD, the term has been reconsidered due to the varied metabolic causes and inconsistent terminology in the literature. Experts now suggest that metabolic (dysfunction)-associated fatty liver disease (MAFLD) is a more accurate term to describe this condition and better classify patients [92]. MAFLD affects 25% of the population, making it the most common liver disease worldwide [92,93,94].

The connection between MAFLD and CVD is not fully understood, but both conditions share several risk factors. Epidemiological studies have found that patients with MAFLD have a higher rate of CVD than the general population. In a 28-year follow-up study of MAFLD patients, CVD was the leading cause of death, with liver disease and cancer being the second-most common causes [94,95,96,97,98]. On the other hand, new evidence has connected NAFLD to an increased occurrence of atherosclerotic disease, attributable to shared risk factors [95].

Recent studies have demonstrated a direct connection between MAFLD and cardiac calcifications, including aortic and mitral valve calcifications, in both diabetic and non-diabetic patients [93,95]. In a cross-sectional study, conducted by Mantovani et al. involving 247 diabetic patients without a history of CVD or liver disease, researchers found that 70.8% had MAFLD. This condition was significantly associated with aortic valve sclerosis and MAC. Additionally, 26.3% of patients had calcifications in either the mitral or aortic valve, while 17.4% had calcification in both valves. The study highlighted a significant association between MAFLD and the presence of either aortic valve calcification or MAC [93,94]. The co-occurrence of aortic valve sclerosis and MAC shows the highest correlation with MAFLD, whereas individuals without any valvular calcification are the least likely to have the disease. Patients with either isolated AVS or isolated MAC have a moderate likelihood. These associations persist regardless of T2D, CKD, any medications, and echocardiographic measurements [92,93,94].

A recent study explored the connection between MAFLD, liver fibrosis score (FIB 4), and MAC due to their links to increased cardiovascular mortality and systemic metabolic syndrome. It involved 100 patients, divided into two groups: those with MAC (26 patients) and those without (74 patients). Analysis showed that individuals with MAC had higher age, serum creatinine levels, MAFLD, and FIB 4 scores, as well as increased rates of hyperlipidemia, T2D, and use of ACE inhibitors and statins. The findings indicate that MAFLD and FIB 4 scores are independently associated with MAC [99].

These studies indicate that calcifications in the mitral and aortic valves are a contributing factor to the higher risk of CVD in patients with MAFLD. However, further research is necessary to explore the relationship between isolated MAC and MAFLD.

### 6.2. Prediabetes and Diabetes Mellitus

Diabetes mellitus is a major cause of illness and early death globally, significantly affecting healthcare systems. Before diabetes develops, individuals experience a condition known as prediabetes, which is linked to an increased risk of serious macrovascular complications such as CVD, stroke, and vascular disease [100]. Important meta-analyses indicate a link between prediabetes and several CVDs, including acute and chronic CAD, heart failure, and atherosclerosis [100,101,102,103].

A prospective population study conducted in the United Kingdom (EPIC-Norfolk study) with 4662 men and 5570 women investigated the relationship between hemoglobin A1c (HbA1c) levels, CVD, and total mortality. The study found that higher HbA1c levels were continuously associated with an increased risk of CVD and mortality, even in individuals without diabetes. Specifically, an increase of 1% in HbA1c was linked to a relative risk of death from any cause of 1.24 in men and 1.28 in women. These associations persisted independent of various risk factors such as age, body mass index, and blood pressure. Most deaths occurred in individuals with HbA1c levels between 5% and 6.9%, rather than those with diabetes. The findings highlight a significant risk associated with elevated HbA1c levels and suggest the need for further randomized trials to explore whether reducing HbA1c could lower cardiovascular risk [104]. Furthermore, another extensive study found comparable results: Compared to men with normal glucose levels, those with known diabetes had a relative risk of death of 2.0, those with newly diagnosed diabetes had a risk of 2.7, and those with impaired glucose tolerance had a risk of 1.6 [105].

In this context, atherosclerotic changes in patients with prediabetes and T2D are influenced by factors such as insulin resistance, abdominal obesity, changes in the serum metabolic profile (like lower HDL-cholesterol level and higher triglycerides level), and HTN, among others [100,106]. It is important to highlight that in the context of atherosclerotic changes, obesity in patients with prediabetes contributes to elevated levels of TNF-α, IL-6, IL-8, and CRP in the bloodstream [100,106].

The link between MAC and T2D has been thoroughly investigated. It is currently observed that MAC is highly prevalent among patients with T2D [57,58,94,107,108]. For example, a recent study found that 42.3% of patients with MAC had T2D, compared to just 17% of those without MAC (*p* = 0.006). Multivariate analysis revealed that patients with T2D were 4.226 times more likely to develop MAC compared to those without T2D [58].

Even more, a study analyzed data from 902 patients with T2D and aimed to evaluate the impact of aortic valve sclerosis and MAC on all-cause and cardiovascular mortality in individuals with T2D. At baseline, 52.9% of patients had no affected valves (HVC-0), 33.7% had one affected valve (HVC-1), and 13.4% had both valves affected (HVC-2). Over a mean follow-up of 9 years, 15.2% of patients died, with 78 of those deaths attributed to cardiovascular causes. Patients with HVC-2 had the highest risk of all-cause and cardiovascular mortality compared to those with HVC-0, while those with HVC-1 had an intermediate risk. After adjusting for various factors, patients with HVC-1 had a 2.3-fold increased risk of all-cause mortality, and those with HVC-2 had a 9.3-fold increased risk. The study concluded that both MAC aortic valve calcification, whether alone or combined, are independently associated with increased mortality in T2D patients [108].

Based on our research for this article, we have not found any studies in the literature to date that examine the presence of MAC in patients with prediabetes. Therefore, further research is needed on this topic, involving a large number of patients with prediabetes, possibly across multiple centers. This would allow for a thorough evaluation of the link between prediabetes and MAC, helping to make early decisions to prevent disease progression and reduce mortality.

### 6.3. Chronic Kidney Disease

Although MAC and CKD share common cardiovascular risk factors, other elements also contribute to the link between these two conditions.

Cardiovascular diseases are the primary cause of death in patients with CKD, with their cardiovascular risk being 20 times higher than that of the general population. This elevated risk persists even in the early stages of CKD. Conditions like HTN, ischemic disease, valvular heart disease, and AF are commonly linked to CKD [109].

Recent studies indicate that the prevalence of MAC in patients with CKD ranges from 5% to 60%. Among dialysis patients, particularly those on hemodialysis for more than three years, the prevalence ranges from 25% to 59% [110]. MAC is about four times more common in patients with renal insufficiency compared to the general population, where its prevalence ranges from 8% to 15%, varying with age and being most common in older individuals. MAC is more frequently observed in end-stage renal disease (ESRD) than in CKD [110].

Although MAC and CKD share common cardiovascular risk factors, other elements also contribute to the link between these two conditions. Several theories explain the mechanisms of MAC in CKD patients, categorized into traditional CVD risk factors, non-traditional risk factors, and CKD-specific factors [111,112,113,114]. Traditional risk factors include advanced age, diabetes, smoking, HTN, lipid metabolism disorders, and obesity, highlighting the shared risk factors for valve and vascular calcification. Non-traditional risk factors involve elevated parathyroid hormone levels, hyperphosphatemia, hypocalcemia, and Vitamin D deficiency. CKD-specific factors include valvular endothelial damage from shear stress, anemia, uremic toxins, and mineral bone disorders. The combination of these factors accelerates MAC in CKD patients [111,112,113,114,115,116].

**Figure 4:**
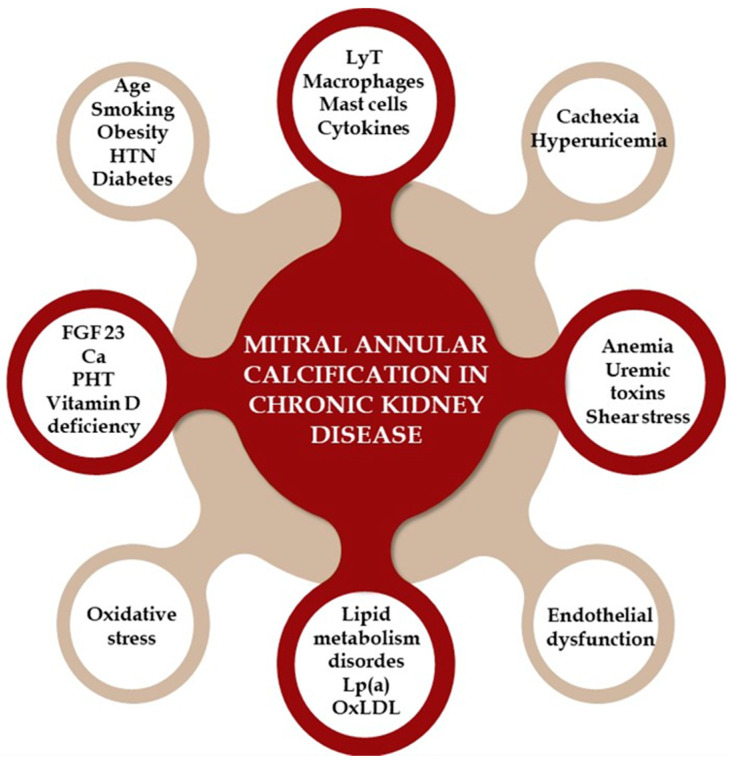
The pathophysiological mechanisms that lead to the development of mitral annular calcification in patients with chronic kidney disease. HTN = hypertension; LyT = T lymphocytes; Lp (a) = lipoprotein (a); OxLDL = oxidized LDL; FGF = fibroblast growth factor; Ca = calcium; PTH = parathyroid hormone.

Chronic inflammation (involving T lymphocytes, macrophages, and cytokines) and increased oxidative stress in calcified valve regions are key contributors to valve calcification in CKD. In early CKD, endothelial dysfunction allows these inflammatory cells and plasma-concentrated molecules, such as oxidized LDL (OxLDL), Lp(a), calcium, phosphorus, and bone metabolism-related molecules like RANKL and FGF23, to infiltrate the mitral and aortic valve, causing thickening. Other proposed mechanisms include malnutrition, cachexia, hyperuricemia, and high sclerostin levels [111,112,114,115,116,117,118,119]. Figure 4 provides a visual representation of the pathophysiological mechanisms that lead to the development of MAC in patients with CKD.

Numerous large studies have documented the association between MAC and CKD. Of the 3047 participants from the Framingham Offspring Study (average age 59, 52% women), 8.6% had CKD. Among those with valvular calcification, 20% had CKD compared to 7% without calcification. After adjusting for various factors, CKD was associated with a 60% higher likelihood of MAC. More than this, CKD combined with MAC significantly increased the risk of death compared to having neither condition [23].

In the Multi-Ethnic Study of Atherosclerosis (MESA), which included ethnically diverse middle-aged adults without obvious CVD, aortic valve calcification and MAC were assessed via CT. Of 6785 participants, 10% had eGFR below 60 mL/min/1.73 m^2^, 15% had diabetes, 13% had aortic valve calcification, and 9% had MAC. For MAC patients, associations with eGFR and cystatin C were significant only in diabetic individuals, with eGFR below 60 mL/min/1.73 m^2^ and higher cystatin C levels strongly linked to MAC in this group. Microalbuminuria was associated with MAC regardless of diabetes status. The study concluded that impaired kidney function has a strong association with MAC, especially in diabetics [120].

In a recent study, a subset of the NEFRONA study sample was randomly selected. This research aimed to assess the prevalence of valvular calcification, track its progression, and examine its associations with various risk factors. The study included 397 patients, with baseline eGFR averaging 33 mL/min, decreasing to 30.9 mL/min over 24 months. There was an increase in carotid and femoral plaque areas and a rise in cases of aortic and mitral valve calcification. MAC at 24 months was positively associated with age, ankle–brachial index (ABI), and calcium–phosphorus product at baseline, but not with eGFR. The study concluded that valvular calcification is prevalent among CKD patients without known CVD and progresses over 2 years regardless of eGFR [109].

Another study conducted by Sharma et al. investigated whether MAC could predict mortality and cardiac disease in renal transplant candidates. The study involved 140 patients who underwent TTE and coronary angiography. Over an average follow-up of 2.2 years, there were 21 deaths. MAC was present in 56 patients (40%) and was linked to higher mortality (*p* = 0.04). Additionally, patients with MAC had higher plasma levels of calcium (*p* = 0.002), phosphate (*p* = 0.004), cardiac troponin T (*p* = 0.03), and N-terminal Pro-B-type natriuretic peptide (*p* = 0.004). Those with MAC had a higher prevalence of T2D (*p* = 0.03), dialysis (*p* = 0.05), significant CAD (*p* < 0.001), and use of calcium-containing phosphate binders (*p* = 0.02) and Vitamin D3 (*p* = 0.04). That being said, MAC is associated with higher mortality in ESRD patients [121]. Above this, MAC was linked to impaired left ventricular function in CKD patients being evaluated for kidney transplantation [122].

### 6.4. Osteoporosis

The occurrence of MAC increases with age and is more frequently observed in women, particularly those who are post-menopausal [123,124,125]. Research indicates that MAC in elderly women, but not in men, can be linked to ectopic calcium deposits resulting from severe bone loss due to postmenopausal osteoporosis [124,125]. Chronic hypercalcemia contributes to the accelerated accumulation of calcium in the annulus and valvular cusps. Additionally, the calcium released from bones is deposited in the mitral valve. The initiation of vascular calcification involves matrix vesicle formation and mineralization, mirroring bone processes. Factors such as oxidized lipids, osteoprotegerin, and bisphosphonates regulate mineralization in bone and vasculature, potentially explaining the simultaneous presence of osteoporosis and vascular calcification [125]. In animal models, bisphosphonate therapy has been shown to reduce valvular calcification. A population-based observational study found that bisphosphonate use was linked to increased valvular and vascular calcification in younger participants but had the opposite effect in older participants [126]. This suggests that bisphosphonates may indicate severe or early-onset osteoporosis in younger individuals, while effectively preventing late-onset, milder osteoporosis in older individuals [126].

Recent research examined 6814 participants (average age 62.2 years; 52.9% female) from the MESA study, all free of cardiovascular disease at baseline. Using non-contrast cardiac CT, MAC was assessed. The median bone mineral density (BMD) was 160.3 mg/cc and was lower in individuals with MAC (132.8 vs. 162.7 mg/cc; *p* < 0.001). After adjusting for bisphosphonate use, MAC was significantly associated with lower BMD (adjusted coefficient: −0.04; 95% CI: −0.06 to −0.02), especially in males (p-interaction: 0.035) [127]. Previous MESA research also indicated that low thoracic BMD correlates with increased aortic valve calcification and MAC [128]. The inverse relationship between cardiac calcification and osteoporosis may be due to tissue responses to chronic inflammation, which promotes bone loss while encouraging calcification in cardiac valves. Common mechanisms in calcium regulation are involved in both atherosclerosis and various forms of vascular calcification [127].

A cross-sectional analysis of 1497 older adults examined the relationship between BMD and calcification of the aortic valve, aortic annulus, and MAC [124]. The study tested the hypothesis that BMD of the total hip and femoral neck, measured by dual-energy X-ray absorptiometry (DEXA), inversely correlates with cardiac calcification prevalence [124]. Results showed that cardiac calcification was prevalent among both women and men (AVC: 59.5% women, 71.0% men; AAC: 45.1% women, 46.7% men; MAC: 42.8% women, 39.5% men). No significant associations were found between continuous BMD and the three calcification measures. Sensitivity analyses suggested potential inverse relationships between femoral neck BMD and AVC with stenosis in men, and femoral neck BMD and moderate/severe MAC in women, though these were not significant [124].

In a prospective observational study conducted at a tertiary referral center by Davutoglu et al., 340 women underwent TTE with an assessment of MAC and BMD measurement using DEXA [125]. Among the participants, 123 patients (first group) had no MAC, while 217 patients (second group) had MAC. The group with MAC showed a significantly higher prevalence and severity of osteoporosis compared to the control group (18.2% vs. 55.5%, *p* < 0.001). Severe osteoporosis was notably more common in the severe MAC subgroup compared to the control subjects (65.2% vs. 17.1%; Pearson χ^2^, 70.02; df = 4; *p* < 0.001). Multivariate analysis revealed that T-scores and age were strong predictors of MAC, with odds ratios of 2.66 (95% CI, 1.85–3.83) and 1.04 (95% CI, 1.01–1.07), respectively. Thus, MAC is linked to osteoporosis, and BMD measurement (T-scores) and age are highly predictive of MAC in women [125].

A cross-sectional analysis of 1497 older adults examined the relationship between BMD and calcification of the aortic valve, aortic annulus, and MAC [124]. The study tested the hypothesis that BMD of the total hip and femoral neck, measured by DEXA, inversely correlates with cardiac calcification prevalence [124]. Results showed that cardiac calcification was prevalent among both women and men (AVC: 59.5% women, 71.0% men; AAC: 45.1% women, 46.7% men; MAC: 42.8% women, 39.5% men). No significant associations were found between continuous BMD and the three calcification measures. Sensitivity analyses suggested potential inverse relationships between femoral neck BMD and AVC with stenosis in men, and femoral neck BMD and moderate/severe MAC in women, though these were not significant [124].

These inconsistent results from studies in the literature highlight the need for further research into sex-specific relationships between low BMD and cardiac calcification, and the potential for targeting these processes therapeutically.

### 6.5. Dementia

Rodriguez et al. conducted research that aimed to explore for the first time the links between MAC, aortic annular calcification, and aortic valve sclerosis with covert brain infarcts detected by MRI [129]. Covert brain infarcts are linked to higher risks of cognitive decline, dementia, and future strokes. The study involved 2680 Cardiovascular Health Study participants, with an average age of 74.5 who underwent brain MRI, followed by TTE. The analysis found that any type of annular or valvular calcification was associated with a higher prevalence of covert brain infarcts (*p* < 0.01). After adjusting for various factors (age, gender, ethnicity, body weight index, exercise level, serum creatinine, systolic blood pressure, total lipid levels, high-density lipoprotein levels, tobacco use, T2D, CAD, and heart failure), the presence of any calcification remained linked to covert infarcts (risk ratio: 1.24; 95% CI: 1.05 to 1.47), with severity correlating with the likelihood of MRI findings [129].

This may be due to factors such as microvasculopathy, edema, gliosis, chronic ischemia, and small infarcts from embolic calcific disease. Although white matter disease and annular or valvular calcification are both common in older adults, their association is unlikely to be mere coincidence. The independent link observed, after accounting for age and other variables, suggests that common risk factors or residual confounding are not the sole explanations [129].

New data from the MESA study found that the incidence of dementia was higher in individuals with MAC (20.4%) compared to those without (7.4%), with rates of 17.9 vs. 5.1 per 1000 person years. Even after adjusting for demographics, cardiovascular risk factors, the presence of coronary artery calcium, and ApoE4 genotype, those with MAC had a significantly higher risk of dementia (adjusted HR: 1.36; 95% CI: 1.10–1.70) [127].

These results suggest that MAC might be a potential indicator of biological brain aging [127].

## 7. Conclusions

In conclusion, MAC represents a complex and evolving aspect of cardiovascular health that extends far beyond its initial classification as a mere age-related phenomenon. This article has elucidated how MAC is now recognized as an active pathological process influenced by systemic inflammation, hemodynamic stress, lipid accumulation, and aberrant bone formation.

The evidence presented underscores that MAC is not just a passive marker of aging but a significant indicator of atherosclerotic burden with profound implications for cardiovascular health. Its correlation with heightened risks of myocardial infarction, stroke, and overall cardiovascular mortality highlights the urgent need for clinicians to consider MAC in the broader context of cardiovascular risk assessment and management.

Through a comprehensive exploration of MAC’s role as a potential embolic source, its diagnostic challenges, and its connections to systemic inflammation and metabolic disorders, this article emphasizes the necessity for continued research and clinical awareness. By advancing our understanding of MAC, we can better address its implications in patient care, refine diagnostic strategies, and develop targeted interventions to mitigate its impact on cardiovascular morbidity and mortality.

## Data Availability

No new data were created or analyzed in this study. Data sharing is not applicable to this article.
